# Use of Caudal *Quadratus Lumborum* Block with Ropivacaine as Part of an Opioid-Free Analgesic Protocol in Dogs Undergoing Orchiectomy: A Randomized Trial

**DOI:** 10.3390/ani14131885

**Published:** 2024-06-26

**Authors:** Andrea Paolini, Roberta Bucci, Amanda Bianchi, Francesca Del Signore, Salvatore Parrillo, Alessandro Di Giosia, Claudia Ristori, Roberto Tamburro, Domenico Robbe, Augusto Carluccio, Valeria Rosa, Massimo Vignoli

**Affiliations:** 1 Small Animal Surgery and Anesthesia Service, Department of Veterinary Medicine, University of Teramo, 64100 Teramo, Italy; apaolini@unite.it (A.P.); abianchi@unite.it (A.B.); cristori@unite.it (C.R.); rtamburro@unite.it (R.T.); valeria.rosa@studenti.unite.it (V.R.); 2 Obstetrics, Gynecology and Veterinary Andrology Clinic Service, Department of Veterinary Medicine, University of Teramo, 64100 Teramo, Italy; rbucci@unite.it (R.B.); alessandrodigiosia551@gmail.com (A.D.G.); drobbe@unite.it (D.R.); acarluccio@unite.it (A.C.); 3 Small Animal Imaging Service, Department of Veterinary Medicine, University of Teramo, 64100 Teramo, Italy; mvignoli@unite.it

**Keywords:** loco-regional anesthesia technique, interfascial plane block, *Quadratus Lumborum* block, ropivacaine, orchiectomy, dogs

## Abstract

**Simple Summary:**

Pain control is challenging in both human and veterinary medicine. The advantages of loco-regional anesthesia are widely known. In the last decade in veterinary medicine, the study of loco-regional interfascial techniques has grown exponentially. The study aims to evaluate the sparing effect on opioid consumption of the *Quadratus Lumborum* block performed at the level of the sixth lumbar vertebra in dogs undergoing elective orchiectomy. The results show an effective reduction in intraoperative consumption of fentanyl in the experimental group receiving the *Quadratus Lumborum* block compared to the control group. No statistically significant difference was found in methadone consumption in the post-operative period. No complications have been reported when performing the *Quadratus Lumborum* block at the level of the sixth lumbar vertebra. The results of this study suggest that the loco-regional anesthesia technique has an opioid-sparing effect on the surgical procedure proposed.

**Abstract:**

In veterinary medicine, the use of loco-regional anesthesia techniques is increasing. The *Quadratus Lumborum* block (QL) is an interfascial loco-regional technique that involves the release of local anesthetic (LA) between the *Quadratus Lumborum* and the *Small Psoas* (Pm) muscle. The study aims to evaluate the effect of the QL block on reducing the total amount of opioids in dogs undergoing pre-scrotal orchiectomy. A group of 36 dogs was enrolled in a randomized blinded study. The animals were divided into two groups: 18 in the experimental group (QL) and 18 in the control group (C). The QL group received 0.4 mL kg^−1^ of ropivacaine 0.5% for each hemiabdomen (total amount of 3 mg kg^−1^ of ropivacaine, 1.5 mg kg^−1^ per side). The C group was brought into the operating room (OR) after receiving the same clipping as the QL group. In the intraoperative period, opioid consumption in the QL group was significantly lower than in the C group. No differences were found in the post-operative phase. No side effects were reported when performing the QL technique. The QL block performed at the level of L6 appears to be a valid approach to reducing opioid use in dogs undergoing orchidectomy with a pre-scrotal surgical approach.

## 1. Introduction

Pain control is challenging in both human and veterinary medicine [1,2,3]. In both humans and animals, the nociceptive stimuli caused by the surgical insult have been shown to lead to several side effects [4,5,6,7]. Adequate analgesic management in the perioperative period leads to the reduction of significant adverse effects, such as lower cellular stress responses and biomarkers, reduction of pain and inflammation cascade activation, pathological pain prevention, and lower recovery time and complications [1,4,8,9,10,11].

For many years, opioids have been a valid approach to the management of perioperative nociceptive stimuli and pain in dogs and cats [12,13]. The reasons for the wide use of opioids are the high therapeutic index, clinical efficacy in visceral, acute, and chronic pain, availability of numerous molecules, the benefit of reversibility, and relatively low costs [13,14]. Sadly, opioids are not without side effects. The repeated use of opioids in veterinary medicine is associated with potential adverse effects, such as depression/lethargy, reduction of gastrointestinal motility, nausea, vomiting, dysphoria, and immunosuppression [15,16,17,18,19]. These complications are associated with a longer recovery time [15,16,17,18,19].

The use of loco-regional anesthesia techniques reduces the use of hypnotic and analgesic drugs, including opioids, and related side effects [4]. One of the great advantages of local anesthetic (LA) use is the blockage of sodium channels in order to prevent the “memory of the nociceptive stimuli and pain” [4]. In recent years, clinical studies on loco-regional anesthesia in dogs have focused on interfascial plane (IFP) techniques such as the *Trasversus Abdominis plane* (TAP) block, *Erector Spinae plane* (ESP) block, the *Serratus plane* (SP) block, and the *Quadratus Lumborum* (QL) block [20,21,22,23,24,25]. The IFP blocks are relatively simple to perform, with a high rate of success. In particular, the QL block is an IFP technique used to provide somatic and visceral analgesia to the abdomen. The QL block stains the ventral rami of the spinal nerves and the sympathetic trunk. This technique was first described in a cadaveric study by Garbin and colleagues [26] in dogs. The study described the QL block at the level of the first lumbar vertebra (L1). Several other cadaveric studies on QL at the L1 level have been published, describing various approaches and different anesthetic injection sites [26,27,28,29,30]. The use of the QL block has an effective sparing effect on systemic analgesia during the perioperative period, including a reduction in opioid consumption [21,24,26,27,28,30]. The QL block is relatively fast and easy to perform, especially when performed by an anesthesiologist with proven experience. The low complication rate and its feasibility were also demonstrated in a clinical study by Viscasillas et al. [31].

The aforementioned features also make the QL block useful in the case of relatively short surgeries, such as orchiectomies. In addition, its effectiveness would be even more advantageous in longer and more demolitive surgeries where the nerve structures of the lumbar and sacral plexus are involved, as in the case of a perineal hernia, for example, where pelvic floor reconstruction is often combined with orchidectomy and colopexy.

The cadaveric study by Otero et al. 2024 [32] described the QL technique performed at the level of the sixth lumbar vertebra (L6) in combination with a greater ischiatic notch (GIN) block to stain the lumbar and sacral plexuses in dogs. To the authors’ knowledge, there are no clinical studies evaluating the efficacy of the QL technique at L6 for orchiectomies in dogs.

The present study aims to evaluate the perioperative analgesic effect of the QL technique at the L6 level in elective orchiectomies with a pre-scrotal approach. The main hypothesis is that the use of the QL technique at L6 reduces opioid consumption in the intraoperative phase; the second hypothesis is that the difference in post-operative methadone requirements is negligible.

## 2. Materials and Methods

### 2.1. Animals

A group of thirty-six male dogs from various breeds who were presented for elective orchiectomy were enrolled in a randomized blinded clinical trial at the Veterinary Teaching Hospital ‘G. Gentile’ (University of Teramo, Italy). Only dogs with ASA status I (American Society of Anesthesiologists) were involved in the study. Other inclusion criteria were a body condition score (BCS) between 3–5, aged between 1–5 years, proper vaccination prophylaxis, and anti-parasite treatments. Exclusion criteria were the presence of any localized comorbidity and/or systemic disease (ASA status ≥2), a BCS <3 and >6/9, aged <1 and >5, and anxious and aggressive or easily stressed dogs who do not allow the placement of a venous catheter while awake. The study was approved by the Scientific Ethics Committee of the University of Teramo, protocol number 12150, with the date 27 April 2023. The owners’ written consent was obtained once the surgical and anesthesiologic procedures were suitably explained. On the day of the surgery, a complete clinical examination and routine exams (blood count and biochemistry) were performed on all dogs. The dogs were accepted and acclimatized in their kennels for 24 h before the procedure. The animals were fasted eight hours before surgery and water was left available until the transfer to the preparation room (OR).

### 2.2. Procedure

The animals were divided into two groups, 18 animals in the control group (C group) and 18 in the *Quadratus Lumborum* group (QL group), with the www.randomizer.org device (access date 7 May 2023). Twenty minutes before premedication, pain assessment (T0) was performed on each dog using the Italian Version of the Glasgow Compositive Measure Pain Scale Short Form (ICMPS-SF) validated by Della Rocca and colleagues [33]. A 20-gauge (G) (Jelco; Smiths Medical, Minneapolis, MN, USA) catheter was applied aseptically into the right cephalic vein. To ensure intraoperative antibiotic prophylaxis, dogs received cefazoline 22 mg kg^−1^ intravenously (IV) (Cefazolina 1 gr, Teva Italia S.r.l., Milan, Italy) 30 min before the surgery [34]. Dexmedetomidine (Dextroquillan 0.5 mg ml; Fatro, Bologna, Italy) 0.002 mg kg^−1^ IV was administered slowly as premedication. Ringer’s lactate solution (Baxter Healthcare Corp, Deerfield, IL, USA) was administered at 5 mL kg^−1^ h. A few minutes after premedication, when the anxiolytic/sedative effect of dexmedetomidine was achieved, flow-by oxygen (150 mL kg^−1^ min^−1^) was administered before induction via an adequate oxygen mask for 3–5 min (SurgiVet Pet Oxygen Masks, Tri-Med Medical Supplies, Inc., Plymouth, UK). The general anesthesia was induced with propofol (Proposure; 10 mg mL, Boehringer Ingelheim Animal Health, Milan, Italia) 2–4 mg kg^−1^ to effect. The trachea was intubated with a PCV tube of the correct size (Rusch, The Sheridan, Morrisville, NC, USA). General anesthesia was maintained with isoflurane (Isoflo; Zoetis, Rome, Italy) delivered in a mixture of 50% oxygen and 50% medical air. Under general anesthesia, a catheter (Jelco; Smiths Medical, Minneapolis, MN, USA) of the correct size was placed in the pedal artery to continuously monitor the arterial pressure. Before the towel clamps were positioned and every 5 min until the end of the surgery, the following vital signs were recorded: oxygen saturation of hemoglobin (SpO_2_); end-tidal isoflurane concentration (EtISO); end-tidal carbon dioxide concentration (EtCO_2_); heart rate (HR); respiratory frequency (*f*R); esophageal temperature (T); and invasive blood pressure (IBP). The abdomen was clipped from the cranial portion of the wing of the ileum and lateral to the spine over the process transverse of L6.

### 2.3. Loco-Regional Anesthesia Technique

**Figure 1:**
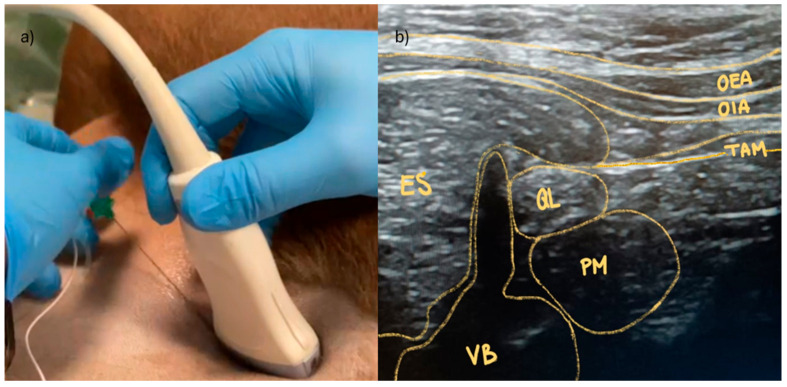
Ultrasound (US)-guided *Quadratus Lumborum* (QL) block at the level of the sixth lumbar (L6) vertebra. (**a**) The dogs were positioned in lateral recumbency. Clipping and surgical scrub at the transverse process of L6 were performed before the block. The needle was introduced with an in-plane approach in a ventro-dorsal direction. (**b**) The US visualization and landmarks of the QL at L6. *Erector spine* muscle (ES); *obliquus external abdominis* muscle (OEA); *obliquus internus abdominis* muscle (OIA); *transverse abdominis* muscle (TAM); *quadratus lumborum* muscle (QL); *psoas minor* muscle (Pm); L6 vertebra.

Dogs were positioned in lateral recumbency and an ultrasound (US)-guided QL block was performed. A high-frequency linear array transducer (7–12 MHz) attached to a US machine (LA523; Esaote, Milan, Italy) was positioned parallel to the cranial portion of the wing of the ileum at the level of the transverse process of L6. The probe marker was oriented dorsally perpendicular to the spine (Figure 1a,b).

All QL blocks were performed by the same veterinarian with proven experience in loco-regional anesthesia (A.P.). After visualization of US landmarks, a 90 mm US-guided needle (US simplex, B. Braun Avitum, Milna, Italy) was introduced with an in-plane approach in the ventro-dorsal direction. The needle was advanced until it reached the area between the QL and Pm muscle strips. If the needle was not visualized correctly or the anesthetic had not spread in the affected area, the needle was repositioned. After negative aspiration and the inoculation of a small test volume of local anesthetic (0.3 mL), ropivacaine 0.5% (Ropivacaine Chloralhydrate, 10 mg mL, Galenica Senese, Siena, Italy) was injected and the correct spread in the affected area was displayed, 0.4 mL kg^−1^ for each side (total amount of 3 mg kg^−1^ of ropivacaine, 1.5 mg kg^−1^ per side). To ensure a blind technique, the same area was clipped on all dogs regardless of the group assigned. Additionally, a patch was placed on the skin of all dogs where the loco-regional needle ideally passes the skin. The operators involved in the study were divided into three work groups: (1) pre-surgical group (A.P., S.P., A.B.) (animals’ preparation and QL block execution); (2) intraoperative assessment and nociceptive stimuli management; (C.R., C.A., F.D.S.); (3) (R.B., V.R., D.R.) post-operative pain and hospitalization assessment. 

### 2.4. Intraoperative Anesthesia Management and Surgical Procedure

After preparation, the dogs were brought into the OR and connected to the workstation to a re-breathing system (Fabius, Draeger, Milan, Italy) and a multiparametric monitoring device (Vista 120s, Draeger, Milan, Italy). Dogs were left in spontaneous ventilation. In case of hypercapnia (end-tidal CO_2_) (EtCO_2_ > 45 mmHg), dogs were mechanically ventilated to maintain normocapnia (EtCO_2_ 35–45 mmHg). The ventilatory setting could be changed by the anesthetist in charge. The following vital signs, HR, *f*R, systolic, mean, and diastolic (SAP, MAP, DAP) with the IBP method, T, EtCO_2_, and EtISO, were recorded before incision into the skin. At the beginning of surgery, the EtISO was set at 1.2. The depth of anesthesia was managed through clinical assessment (eyelid reflex, jaw tone, absence of movement, and ventrally rotated eyes) and set according to the anesthetist in charge of the dogs’ needs. 

In case of hypotension (<60 mmHg of MAP), the anesthesia depth was lightened, and at the same time, the anesthetist chose whether to administer one or more of the following drugs: (1) a bolus of crystalloids (Ringer Lactate solution, Baxter Healthcare Corp, Deerfield, IL, USA) at 10 mL kg^−1^ during 20 min IV; (2) anticholinergic drugs (atropine, Atropine sulfate 10 mL, 1 mg mL, ATI, Bologna, Italy) at a dose of 0.02–0.04 mg kg^1^ IV or vasopressors such as noradrenaline (noradrenaline sulfate 2 mg mL, Galenica Senese, Siena, Italy), initial dose 0.0001 mg kg^−1^ min^−1^ IV, increasing slowly to effect up to a maximum of 0.001 mg kg^−1^ min^−1^. Any type of changes in the ventilatory setting or treatments administered were reported on the anesthetic record. In case of an increase of more than 20% in HR and/or *f*R and/or SAP above baseline values, rescue analgesia was given. A bolus of 0.002 mg kg^−1^ of fentanyl (Fentadon; Eurovet Animal Health, Bladel, The Netherlands) was administered. In case of a nociceptive reaction, a second bolus of fentanyl at the same dose was administered followed by CRI of 0.005 mg kg^−1^ h^−1^. Nociceptive intraoperative stimuli were divided into skin incision (NS1), first testis removal, (NS2), and second testis removal (NS3). The total amount of intraoperative fentanyl consumption was recorded. The surgical procedures were performed by the same two surgeons (A.D.G. and A.C.). Dogs were positioned in dorsal recumbency; after aseptic preparation, a prescrotal 3–4 cm skin incision was performed. The testicle was exposed by blunt dissection and the vaginal tunic was incised to exteriorize the testicle. The spermatic cord was ligated and transected to remove the testicle. Subcutaneous tissue and skin were routinely closed. Surgical times were recorded. All dogs were evaluated for pain before surgery (T0) and T1, T2, T4, T6, and T8 h after the end of surgery through ICMPS-SF. In case of pain (6 ≤ 24), the dogs received methadone (Semfortan; Eurovet Animal Health, Bladel, The Netherlands) slowly via the IV route at 0.2 mg kg ^1^. In case of side effects such as dysphoria, sialorrhea, and vomiting, the dogs were treated. After 8 h post-surgery, the dogs received meloxicam 0.2 mg kg^−1^ IV (Meloxidolor; Le Vet Beheer, Oldeholtpade, The Netherlands) before being discharged.

### 2.5. Statistical Analysis

Data analyses were performed using R and the following packages: ggpubr and psych (R-4.3.3 for Windows version). The data distribution was assessed using the D’Agostino and Pearson tests. Mean and standard deviation (SD) were used for normality data distribution, whereas non-normally distributed data were expressed as median and range. A priori analysis based on previously published data by [24] was performed for sample size calculation. The sample size effect was estimated to be 0.7, assuming a probability (power) of 0.8 and *α* of 0.05. Intraoperative pain scores and rescue analgesia were chosen as the interest of effect. The Mann–Whitney U test was used to compare age, weight, surgical time, pre-incisional HR, pre-incisional *f*R, pre-incisional SAP, MAP and DAP, pre-incisional T, and EtISO. Fisher’s exact test was used to compare intra and postoperative opioid requirements and pain scores at each time point. Statistical significance was set at *p* < 0.05.

## 3. Results

**Figure 2:**
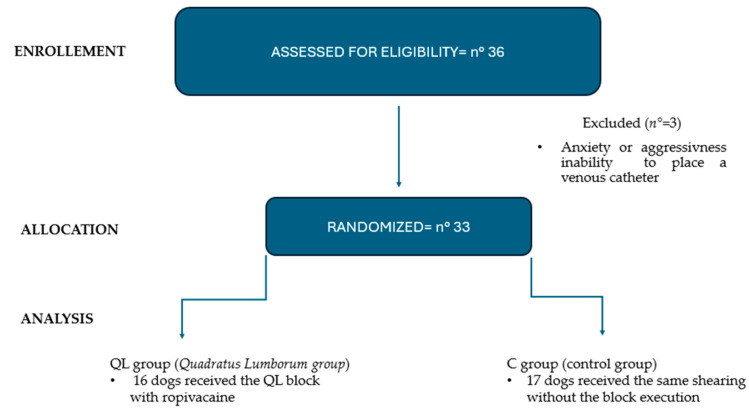
Flow chart diagram.

Thirty-six dogs were recruited for this study from 5 May 2023 to 7 July 2023 and divided into two groups. Three dogs (two from the C group and one from the QL group) were excluded due to the impossibility of placing venous access while they were awake. The following consort flowchart demonstrates the animals’ recruitment and progress (Figure 2). 

No differences were found between demographic and pre-incisional data. These results are displayed in Table 1.

No dogs demonstrated hypoventilation and hypotension during surgical procedures, so no dogs received mechanical ventilation and/or treatment for the management of hypotension. 

Regarding total intraoperative rescue analgesia, dogs in the QL group received 5 (1–5) boluses of fentanyl, less than the C group with 28 (11–28) boluses (*p* < 0.001). In particular, in the C group, fentanyl was administered 19/28 times because of reactions to the skin incision, and the remaining nine times because of testicular reaction. The QL group required 3/4 rescue analgesia for the skin incision and one for the testis. When comparing skin and testis rescue analgesia in the C versus QL groups (19 vs. 2 and 9 vs. 1, respectively), the statistical difference is strongly significant (*p* = 0.015 for the skin and *p* < 0.01 for the testis). The results are shown in Table 2 and Table 3.

In the post-operative period, no differences were observed between groups for the total amount of required methadone. In the C group, 4/16 dogs were treated at the first time point (T1), while 2/17 dogs in the QL group received methadone at T1 (*p* = 0.67) (See Table 3). No animals in either group received methadone from T2 to T8. No side effects such as dysphoria, sialorrhea, and vomiting were present in dogs of either the QL or C group.

## 4. Discussion

Based on the results of this study, the use of the *Quadratus Lumborum* (QL) block performed at the level of L6 decreases the total opioid consumption in dogs undergoing elective orchiectomy with pre-scrotal incisions. The data show a significant reduction of total perioperative opioid demand in the QL group compared to the C group. Our results are in agreement with the main goals of loco-regional anesthesia: less consumption of systemic analgesia (in particular opioids), hemodynamic and respiratory stability, lower immune depression, lower post-operative pain scores, and fewer complications in the post-operative period with a reduction in recovery time [8,35,36,37]. 

### 4.1. Intraoperative Considerations

Several clinical studies on the use of IFP techniques also seem to agree with the findings of this study [20,21,22,23,24]. To the authors’ knowledge, this is the first clinical study on the use of the QL block performed at the L6 level to provide analgesia in dogs undergoing elective orchiectomies. Taking into account the pre-scrotal surgical approach, the structures involved are the skin between the scrotum and penis, the tunica dartos, the cremaster muscle, the epididymis, and the testes. The somatic and visceral components that innervate these structures originate mainly from the lumbar and sacral plexes [38]. The cremaster muscle, epididymis, and testicles are largely innervated by the somatic and visceral components of the sympathetic trunk of the L4–L6 segments, whereas the pre-scrotal skin and tunica dartos appear to be predominantly innervated by the sacral segments of S1–S3 [38]. These anatomical considerations could explain the trend of the results. In the four cases treated intraoperatively with rescue analgesia in the QL group, three were for skin reactions and one was for a testicular reaction. This suggests that there was more effective coverage of the lumbar plexus than the sacral one. The testis reaction in one dog could be due to the partial or total failure of the block due to incorrect and/or uneven distribution. In addition to the possible partial coverage of the block, the high concentration of skin receptors sometimes makes it a real challenge for nociceptive management [39,40]. Therefore, it is also possible to assume that the proposed spread (0.4 mL kg^−1^) desensitizes the lumbar plexus but only partially desensitizes the sacral plexus. Regarding the QL block performed at L1, more studies evaluated different injection spreads using several approaches [26,27,28,29,30,41]. Overall, the literature is in agreement on the anesthetic stain, regardless of the volumes used. In the aforementioned references, volumes ranging from 0.3 up to 0.6 mL kg^−1^ are discussed. In these cases, the block resulted in a substantial staining of the ventral rami of the spinal nerve between the thirteenth thoracic (T13) and fourth lumbar (L4) vertebra and between the first (L1) and third (L3) lumbar vertebra. No cadaveric or clinical studies are available in the literature that have investigated the anesthetic spread, concentration, and volume of ropivacaine for the QL block at the level of L6. In a cadaveric study, the QL block at L6 is described with a spread of 0.3 mL kg^−1^ [32]. Unfortunately, it is not possible to compare our results for different reasons. Firstly, QL is combined with the GIN block to increase the spread. The decision to combine the two techniques resulted in overlapping anesthetic volumes. It is conceivable that the two techniques combined increase the spread caudally, but it is unknown how caudal the QL block may have been. Indeed, despite a correct visualization in the area of the anesthetic, it was not possible to verify the cranial and/or caudal spread concerning the injection site (L6). Regardless of the anesthetic spread, the systemic absorption of ropivacaine must be taken into consideration when analyzing the efficacy. To date, the hypothesis of the systemic absorption of the LA when performing a peripheral or interfascial loco-regional technique is debated [42,43,44,45]. The LA released in the retroperitoneal area (between the psoas minor and QL muscles) has a systemic absorption component. This may concern the analgesia provided through systemic absorption and may have played a significant role in case the spread of the anesthetic is not sufficiently caudal to the sacral plexus in the QL group.

### 4.2. Post-Operative Considerations

Regarding the post-operative period, no significant differences were found between the groups. Several loco-regional clinical studies on IFP blocks are in agreement with the results of this study [20,21,22,23,24,46]. There could be several reasons for these results. In these studies, the opioid-sparing effect is significant only intraoperatively and not post-operatively. In fact, the data show the same benefits in the post-operative period regardless of whether the blockade is performed. This is partly attributable to the fact that the study designs involve elective surgery for which the most complex nociceptive stimulus to manage is in the intraoperative period. The analgesic power of the drugs administered as premedication, which continue to interact throughout the perioperative period, must also be taken into account. Finally, pain in the post-operative period in dogs undergoing minimally invasive elective surgeries such as ovariectomies (OV) and orchiectomies with reduced laparotomic incisions or laparoscopic surgeries appears to be reduced [47,48,49,50]. In any case, the authors hypothesize that differences in pain management may instead be more marked in future studies of the application of this technique for more invasive surgeries, such as perineal hernia, where orchidectomies, colopexies, and pelvic floor reconstructions are involved. 

The correct use of these techniques prevents the surgical insult of Na^+^ channel blockage [35,36,37]. The early blockage of the pain pathway interrupts the nociceptive stimuli to the spinal cord, thus reducing pain perception in the intra and post-operative period [35,36,37]. The ropivacaine concentration of 0.5% was chosen as a compromise between clinical effect, staining, and duration of action. In addition, ropivacaine proposed at this concentration can provide up to 6–8 h of analgesia [45,51]. For this reason and so that there was no interference with post-operative pain assessment, the authors decided to administer an anti-inflammatory drug (NSAID) after the evaluation of the pain score at T 8. A total of six dogs, four in the C group and two in the QL group, received methadone in the post-operative phase. The results are not significant; however, all administrations occurred at T1 (the first post-extubation evaluation). All the animals treated in the post-operative period also received intraoperative fentanyl (animals in both the C and QL groups). This can be partly explained by the fact that the dogs with a positive pain score received methadone, an opioid, which may have covered the pain until the end of the pain score evaluations or until pain perception was reduced. On the other hand, not all dogs treated intraoperatively with fentanyl then needed methadone post-operatively. Another explanation for the lack of difference in post-operative pain may lie in the fact that correct systemic analgesia results in a good outcome in the first post-operative pain assessment. In any case, this consideration does not detract from the benefits of the use of loco-regional anesthesia. In fact, faster and better hospitalization is widely demonstrated. It is reasonable to assume that longer and more complex surgery (for example, perineal hernia) will show more changes in pain assessment in the post-operative period. In any case, fewer dogs received intra and post-operative treatment in the QL group. In this case, the systemic absorption of the anesthetic may have assisted the analgesia, even if it is not administered in the correct IFP space. No side effects such as vomiting, diarrhea, and dysphoria were observed among the dogs involved and all animals were discharged at the end of the study (8 h post-surgery).

This study has limitations. The number of animals included in the study is limited, although a power study was conducted with a sample size calculation. The sample size was calculated based on total opioid consumption in the intraoperative phase of QL performed at L1 and this may have interfered with the different approach and with the results of methadone consumption in the post-operative period. Furthermore, no cadaveric studies are present to indicate the volumes and relative spread of the QL at the level of the sixth lumbar vertebra. In this case, the rationale for the study is linked to the anatomical knowledge of the dog and proven clinical experience in the use of local-regional anesthesia techniques. This led the authors to choose volumes and concentrations of the LA to guarantee analgesic efficacy and a satisfactory duration of action.

## 5. Conclusions

The QL block performed at L6 appears to significantly reduce the total amount of opioids in dogs undergoing orchiectomy with a pre-scrotal surgical approach with respect to the control group. Further studies are needed to evaluate the effective spread of 0.4 mL kg^−1^ of local anesthetic at the L6 level. No complications were reported when performing the QL block.

## Figures and Tables

**Table 1:** Pre-incisional basal values and demographic data in the control (C) and *Quadratus Lumborum* block groups (QL). Weight, age, surgical end-tidal isoflurane (EtISO), pre-incisional heart rate (HR), systolic, mean, and diastolic pressure (SAP, MAP, and DAP), and Temperature (T) are presented as the mean and standard deviation, while surgical time and respiratory rate (*f*R) are presented as the median and range.

Variable	C Group (*n* = 16)	QL Group (*n* = 17)	*p*
Weight (kg) Age (months)	14.9 ± 3.07 27 ± 7	15.01 ± 3.69 32 ± 3	0.67 0.45
Surgical time (min)	15 (13–20)	16 (15–22)	0.81
Surgical EtISO (%)	1.1 ± 0.1	1.0 ± 0.2	0.95
Pre-incisional HR (bpm)	104 ± 15	109 ± 14	0.77
Pre-incisional *f*R (breaths per min) Pre-incisional SAP (mmHg)	20 (16–24) 117 ± 17	20 (16–24) 109 ± 15	1.00 0.88
Pre-incisional MAP (mmHg) Pre-incisional DAP (mmHg)	88 ± 16 55 ± 11	80 ± 12 53 ± 9	0.77 0.89
Pre-incisional T (°C)	38.9 ± 0.18	38.8 ± 0.20	0.84

**Table 2:** Fentanyl requirements as rescue analgesia in the two groups for skin incision and testis.

Variable	C Group (*n* = 16)	QL Group (*n* = 17)	*p*
Rescue analgesia skin incision	19	3	0.015
Rescue analgesia testis	9	1	<0.01

**Table 3:** Total amount of opioids received in intra and post-operative periods.

Variable	C Group (*n* = 16)	QL Group (*n* = 17)	*p*
Total Intraoperative fentanyl (μg kg^−1^)	28 (11–28)	4 (1–4)	<0.001
Post-operative methadone (mg kg^−1^)	4 (0–4)	2 (0–0.2)	0.67

## Data Availability

The data generated in this study are already added in the tables of this article. If you need any further information, please feel free to contact the authors.
